# A Critical Review of Biomarkers Used for Monitoring Human Exposure to Lead: Advantages, Limitations, and Future Needs

**DOI:** 10.1289/ehp.7917

**Published:** 2005-08-11

**Authors:** Fernando Barbosa, José Eduardo Tanus-Santos, Raquel Fernanda Gerlach, Patrick J. Parsons

**Affiliations:** 1 Department of Pharmacology, Faculty of Medicine of Ribeirão Preto, University of São Paulo, Ribeirão Preto, Brazil; 2 Department of Morphology, Estomatology and Physiology, Dental School of Ribeirão Preto, University of São Paulo, Ribeirão Preto, Brazil; 3 Wadsworth Center, New York State Department of Health, Albany, New York, USA

**Keywords:** biomarkers, biomonitoring, blood, bone, feces, hair, lead, plasma, saliva, teeth, toxicokinetics, urine

## Abstract

Lead concentration in whole blood (BPb) is the primary biomarker used to monitor exposure to this metallic element. The U.S. Centers for Disease Control and Prevention and the World Health Organization define a BPb of 10 μg/dL (0.48 μmol/L) as the threshold of concern in young children. However, recent studies have reported the possibility of adverse health effects, including intellectual impairment in young children, at BPb levels < 10 μg/dL, suggesting that there is no safe level of exposure. It appears impossible to differentiate between low-level chronic Pb exposure and a high-level short Pb exposure based on a single BPb measurement; therefore, serial BPb measurements offer a better estimation of possible health outcomes. The difficulty in assessing the exact nature of Pb exposure is dependent not so much on problems with current analytical methodologies, but rather on the complex toxicokinetics of Pb within various body compartments (i.e., cycling of Pb between bone, blood, and soft tissues). If we are to differentiate more effectively between Pb stored in the body for years and Pb from recent exposure, information on other biomarkers of exposure may be needed. None of the current biomarkers of internal Pb dose have yet been accepted by the scientific community as a reliable substitute for a BPb measurement. This review focuses on the limitations of biomarkers of Pb exposure and the need to improve the accuracy of their measurement. We present here only the traditional analytical protocols in current use, and we attempt to assess the influence of confounding variables on BPb levels. Finally, we discuss the interpretation of BPb data with respect to both external and endogenous Pb exposure, past or recent exposure, as well as the significance of Pb determinations in human specimens including hair, nails, saliva, bone, blood (plasma, whole blood), urine, feces, and exfoliated teeth.

Over the last two decades, atmospheric concentrations of lead have decreased significantly around the globe as more and more nations have chosen to remove tetraethylead from gasoline (Thomas et al. 1999). However, humans may also be exposed to Pb through contaminated food, water, and house dust and through industrial activities such as metal recycling and the battery industry. In the United States, for example, although the use of Pb in house paint peaked in 1940 and was banned in 1978, 40% of the nation’s housing stock is estimated to still contain Pb-based paint (Wakefield 2002).

After Pb enters the body, it can travel along several pathways depending on its source and, by extension, its bioavailability. The fraction of Pb that is absorbed depends mainly on the physical and chemical form, particularly particle size and the solubility of the specific compound. Other important factors are specific to the exposed subject, such as age, sex, nutritional status and, possibly, genetic background [Agency for Toxic Substances and Disease Registry (ATSDR) 1999; National Research Council 1993]. One of the earliest toxicokinetics studies reported that Pb, once absorbed into the blood compartment, has a mean biological half-life of about 40 days in adult males (Rabinowitz et al. 1976). The half-life in children and in pregnant women was reported to be longer, because of bone remodeling (Gulson et al. 1996; Manton et al. 2000). However, another study was unable to confirm this finding (Succop et al. 1998).

Like many other “bone-seeking” elements, Pb from blood is incorporated into calcified tissues such as bone and teeth, where it can remain for years (Rabinowitz 1991; O’Flaherty 1995). According to Rabinowitz (1991), the half-life of Pb in bone (bone-Pb) ranges from 10 to 30 years. However, the use of the term “half-life” to describe the biological clearance of Pb from bone implicitly makes assumptions about the kinetics of the process by which Pb is released. Some researchers prefer to use the term “residence time” to avoid implying more precision than what can be directly determined (Chettle D, personal communication). From calcified tissue stores, Pb is slowly released, depending on bone turnover rates, which in turn are a function of the type of bone, whether compact (slow turnover) or trabecular (rapid turnover) (O’Flaherty 1995). Brito et al. (2002) reported that the release rate of Pb from bone varies with age and intensity of exposure. Brito et al. (2005) also examined estimates of exchange rates among compartments. The transfer of Pb from blood to other compartments was much more rapid than the 1-month estimate reported previously (Brito et al. 2005), with the overall clearance rate from blood (sum of rates from blood to cortical bone, to trabecular bone and to other tissue), implying a half-life of 10–12 days (Brito et al. 2005). This highlights the difference between the overall clearance viewed from outside, when no allowance can be made for recirculation, and actual transfer rates.

Physiologic differences between children and adults account for much of the increased susceptibility of small children to the deleterious effects of Pb: whereas in adults 94% of Pb body burden is stored in bones and teeth, this proportion is only 70% in children (Barry 1981). In addition, the continuous growth of young children implies constant bone remodeling for skeletal development (O’Flaherty 1995). This contributes to a state in which Pb stored in bone is continually released back into the blood compartment, a process that has been described as “endogenous contamination” (Gulson et al. 1996). This process is particularly significant for pregnant women because pregnancy causes an increase in bone remodeling. The apparently limited success of various Pb hazard control measures in decreasing blood Pb (BPb) levels in exposed children and pregnant women may reflect a constant bone resorption process (Rust et al. 1999). Popovic et al. (2005) recently reported very different long-term Pb kinetics between men and women, with premenopausal women appearing to retain Pb more avidly or release Pb more slowly compared to postmenopausal women and to men.

## Biomonitoring Human Exposure to Lead

Biomonitoring for human exposure to Pb reflects an individual’s current body burden, which is a function of recent and/or past exposure. Thus, the appropriate selection and measurement of biomarkers of Pb exposure is of critical importance for health care management purposes, public health decision making, and primary prevention activities.

It is well known that Pb affects several enzymatic processes responsible for heme synthesis. Lead directly inhibits the activity of the cytoplasmic enzyme δ-aminolevulinic acid dehydratase (ALAD), resulting in a negative exponential relationship between ALAD and BPb. Pb depresses coproporphyrinogen oxidase, resulting in increased coproporphyrin activity. Pb also interferes with the normal functioning of the intramitochondrial enzyme ferrochelatase, which is responsible for the chelation of iron by protoporphyrin. Failure to insert Fe into the protoporphyrin ring results in depressed heme formation and an accumulation of protoporphyrin; this in turn chelates zinc in place of Fe, to form zinc protoporphyrin. These effects also result in modifications of some other metabolite concentrations in urine (ALA-U), blood, (ALA-B) and plasma (ALA-P), coproporphyrin in urine (CP). The activities of pyrimidine nucleotidase (P5’N) and nicotinamide adenine dinucleotide synthase (NADS) are also modified in blood after Pb exposure. Levels of these various metabolites in biological fluids have been used in the past to diagnose Pb poisoning when direct Pb levels were difficult to obtain in tissues or body fluids (Leung et al. 1993) or as information complementary to BPb test results. They are more accurately described as biomarkers for toxic effects of Pb. In this review we focus on markers that are more accurately defined as biomarkers of Pb exposure, namely, Pb concentrations in biological tissues and fluids. Biomarkers for the toxic effects of Pb have been reviewed in some detail elsewhere (Sakai 2000).

Throughout the last five decades, whole blood has been the primary biological fluid used for assessment of Pb exposure, both for screening and diagnostic purposes and for biomonitoring purposes in the long term. Although BPb measurements reflect recent exposure, they may also represent past exposures, as a result of Pb mobilization from bone back into blood (Gulson et al. 1996). In those subjects without excessive exposure to Pb, 45–75% of the Pb in blood may have come from bone (Gulson et al. 1995; Smith et al. 1996). In exposed children, however, it has been reported that the bone-Pb contribution to blood can be 90% or more (Gwiazda et al. 2005). Thus, reductions in BPb levels after environmental Pb remediation may be buffered somewhat by contributions from endogenous Pb sources (Lowry et al. 2004; Rust et al. 1999). Remediation efforts typically result in reductions of BPb levels in exposed children of no more than 30%, when evaluated within several months after intervention (U.S. Enviromental Protection Agency 1995). Roberts et al. (2001) reported that in children with BPb levels between 25 and 29 μg/dL who were not treated with chelation drugs, the time required for BPb to decline to < 10 μg/dL is about 2 years. Some researchers have suggested that the efficacy of Pb hazard remediation efforts should be evaluated over extended periods to allow adequate time for mobilization and depletion of accumulated skeletal Pb stores and to allow a reduction in the absolute contribution to BPb levels from these stores (Gwiazda et al. 2005; Lowry et al. 2004). Thus, the mean of serial BPb levels should be a more accurate index of long-term Pb exposure.

Data collected as part of the U.S. National Health and Examination Survey (NHANES) give the 95th percentile for BPb as 7.0 μg/dL for children 1–5 years of age and as 5.20 μg/dL for adults 20 years of age and older [U.S. Centers for Disease Control and Prevention (CDC) 2003]. Although the BPb levels of U.S. populations have dropped markedly compared to 30 years ago, new concerns have been raised regarding possible adverse health effects in children at BPb levels < 10 μg/dL; perhaps there is no safe threshold but, rather, a continuum of toxic effects (Canfield et al. 2003). In light of these concerns, the CDC Advisory Committee on Childhood Lead Poisoning Prevention formed a working group to review the evidence for adverse health effects at BPb levels < 10 μg/dL in children. Although this working group concluded that several studies in the literature had demonstrated a statistically significant association between BPb levels < 10 μg/dL and some adverse health effects in children, the effects were very small and could conceivably have been influenced by residual confounding factors. The working group’s report called for further studies to examine the relationship between lower BPb levels and health outcomes to provide a more complete understanding of this issue (CDC 2004).

Many studies have reported statistically significant associations between BPb levels and various health effect outcomes. Some, however, have been statistically weak, with the magnitude of the effect relatively small. According to Hu et al. (1998), such weaknesses of association may occur because BPb is not a sufficiently sensitive biomarker of exposure or dose at the target organ(s) or because the relationships involved are biologically irrelevant and are only found because of an uncontrolled confounding factor. Furthermore, in view of the kinetics of Pb distribution within the body (cycling among blood, bone, and soft tissues), differentiation of low-level chronic exposure from a short high-level exposure is not possible on the basis of a single BPb measurement (Hu et al. 1998). Consequently, there is renewed interest in alternative biomarkers that may aid diagnosis of the extent of Pb exposure. Such alternatives include Pb determinations in plasma/serum, saliva, bone, teeth, feces, and urine. However, none of these matrices has gained convincing acceptance as an alternative to BPb. This is partly due to data based on erroneous or dubious analytical protocols that do not consider the confounding variables.

## Plasma/Serum Lead

Plasma-Pb likely represents a more relevant index of exposure to, distribution of, and health risks associated with Pb than does BPb. Indeed, from a physiologic point of view, we can assume that the toxic effects of Pb are primarily associated with plasma-Pb because this fraction is the most rapidly exchangeable one in the blood compartment. In recent years increased attention has been paid to monitoring the concentration of Pb in plasma (or serum). However, research on associations between plasma-Pb and toxicologic outcomes is still sparse, and a significant gap in knowledge remains.

Plasma/serum Pb levels in nonexposed and exposed individuals reported in older publications range widely, from 0.02 to 14.5 μg/L (Versieck and Cornelis 1988). This is probably due to inappropriate collection methods, analytical instrumentation, and methods for Pb determination. The development and use of more sensitive analytical instrumentation, especially inductively coupled plasma mass spectrometry (ICP-MS), has resulted in determinations of Pb in plasma and serum specimens with much lower detection limits and with better accuracy. More recent data, also based on ICP-MS methods, have shown plasma-Pb levels < 1.0 μg/L in nonexposed individuals (Schutz et al. 1996).

The use of advanced analytical techniques is not the only essential requirement to ensure accurate and reliable plasma-Pb data. Contamination of the specimen may occur at the preanalytical phase, namely, during collection, manipulation, or storage. Use of Class-100 biosafety cabinets and clean rooms for specimen preparation and analysis is mandatory. Moreover, all analytical reagents used must be of the highest purity grade. These conditions are far more rigorous than are typically required for clinical BPb measurements performed in a commercial laboratory. After the blood specimen has been collected, the serum/plasma separation must be performed as soon as possible because there is high potential for Pb to move from the dominant BPb subcompartment repository, namely, the erythrocytes, into the plasma via hemolysis, leading to erroneously high results for plasma-Pb. Plasma hemolysis can be estimated by analyzing hemoglobin levels in the specimen because these levels are likely to become abnormally elevated with hemolysis (Smith et al. 2002). Materials for specimen collection and storage and the anticoagulant must be of the highest quality because these can be another source of Pb contamination.

Commercial evacuated blood tubes, prepared specifically for BPb measurements, are available with < 5 μg/L Pb (Esernio-Jenssen et al. 1999), but it is nevertheless desirable for the analyzing laboratory to characterize the background Pb contamination in each new lot of tubes to ensure that reported concentrations are not compromised by contamination. The choice of anticoagulant is important because EDTA, as a strong metal-chelating agent, may be difficult to obtain without some background contamination and may give misleadingly high plasma-Pb results because of selective extraction of Pb bound to erythrocytes. The use of heparin is problematic because heparinized blood is more prone to form fibrin clots after several hours. These issues were evaluated by Smith et al. (1998) in some detail; they compared commercial Vacutainer-type tubes with ultracleaned collection tubes containing either EDTA or heparin. As there are no commercial blood collection tubes available that are certified for ultra-low Pb measurements, the analyzing laboratory should prepare precleaned polyethylene tubes containing ultra low-Pb anticoagulants.

There are many reports of plasma-Pb measurements where validation data are either weak or absent. For example, some simply cite successful participation of the analyzing laboratory in quality assurance (QA) programs for whole blood Pb operated by the CDC and the College of American Pathologists (Hernandez-Avila et al. 1998), whereas others neglect to cite any kind of QA program (Dombovari et al. 2001). Participation in QA schemes designed specifically for whole BPb, while commendable, does not address the much more challenging analysis for plasma-Pb. This problem is compounded by the lack of certified reference materials for either serum or plasma-Pb (Cake et al. 1996). For these reasons, production of plasma or serum reference materials that have Pb concentrations certified close to current human values is urgently needed to support method validation.

## Saliva Lead

Saliva has been proposed as a diagnostic specimen for various purposes, as it is easily collected (Silbergeld 1993). However, in the absence of consistent and dependable saliva Pb measurements, it is not generally accepted as a reliable biomarker of Pb exposure. Saliva shows large variations in its ion content throughout the day, coupled with changes in salivary flow rates before, during, and after meals. Variations also arise depending on the manner in which saliva collection is stimulated (or not) and on the nutritional and hormonal status of the individual.

Some data suggest an association between Pb levels in saliva and those in either plasma or blood (Omokhodion and Crockford 1991; Pan 1981). Moreover, it has been argued that Pb in saliva is the direct excretion of the Pb fraction in diffusible plasma namely, the fraction not bound to proteins) (Omokhodion and Crockford 1991). Despite the associations reported in the literature, the older saliva Pb concentrations are quite high, and the values vary among studies. Recent data suggest much lower saliva Pb levels, in both exposed and unexposed subjects (Koh et al. 2003; Wilhelm et al. 2002). According to Wilhelm et al. (2002), Pb content in the saliva of unexposed children is usually < 0.15 μg/dL.

Uncontrolled variation in salivary flow rates, lack of standard or certified reference materials, and absence of reliable reference values for human populations are major factors that limit the utility of saliva Pb measurements. In addition the very low levels of Pb present in saliva limit the range of suitable analytical techniques, thereby further diminishing the utility and reliability of this biomarker for evaluating Pb exposure.

## Hair Lead

Hair is a biological specimen that is easily and noninvasively collected, with minimal cost, and it is easily stored and transported to the laboratory for analysis. These attributes make hair an attractive biomonitoring substrate, at least superficially. Because Pb is excreted in hair, many have suggested it for assessing Pb exposure, particularly in developing countries where specialized laboratory services may be unavailable and resources are limited (Schumacher et al. 1991). However, an extensive debate is ongoing about the limitations of hair as a biomarker of metal exposure generally. Here we limit our discussion to Pb exposure, although many of the issues for Pb, such as preanalytical concerns for contamination control, sampling, and reference ranges, also apply to other metals.

The ability to distinguish between Pb that is endogenous, namely, absorbed into the blood and incorporated into the hair matrix, and Pb that is exogenous, namely, derived from external contamination, is a major problem. During the washing step it is assumed that exogenous Pb is completely removed, whereas endogenous Pb is not. However, no consensus exists about how removal of exogenous Pb is best accomplished. Some publications that describe the use of hair for assessing Pb exposure reference a hair washing method proposed by the International Atomic Energy Agency (IAEA) in 1978. The approach entailed washing hair specimens with acetone/water/acetone (Ryabukin 1978). However, a recent study (Morton et al. 2002) demonstrated that the IAEA method failed to remove exogenous Pb from hair.

Another issue is the significant variation in the Pb concentration profile among various subpopulations according to age, sex, hair color, and smoking status (Wolfsperger et al. 1994). Moreover, geographic, racial/ethnic, and ecologic factors can also affect Pb distribution in hair within a given population. Thus, it is difficult to establish reference ranges because confounding factors impose restrictions on the interpretation of individual results. No consensus exists on the length of the hair specimen to be collected, or the amount, or the position on scalp. Variations in Pb content between single hairs from the same individual can be as high as ± 100%, particularly in the distal region (Renshaw et al. 1976).

Recently, the ATSDR established an expert advisory panel to review current knowledge about the use of hair analysis for trace metals in biomonitoring (ATSDR 2001). The general consensus was that many scientific issues need to be resolved before hair analysis can become a useful tool in understanding environmental exposures. Although hair analysis may be able to answer some specific questions about environmental exposure to a few substances, it often raises more questions than it answers. The scientific community currently does not know the range of Pb contamination levels typically found in human hair. Without reliable data on baseline or background hair contamination levels in the general population, health agencies cannot determine whether results from a given site are unusually high or low (ATSDR 2001).

In addition to the preanalytical issues and the absence of reliable reference ranges, the quality of analytical techniques used for determining Pb, as well as other trace metals, in hair has been questioned. In a recent interlaboratory study of commercial laboratories that specifically market the test for trace metals in hair, interlaboratory agreement was judged very poor, with wide discrepancies observed for Pb as well as for other elements (Seidel et al. 2001).

## Urinary and Fecal Lead

The determination of Pb in urine (urine-Pb) is considered to reflect Pb that has diffused from plasma and is excreted through the kidneys. Collection of urine for Pb measurements is noninvasive and is favored for long-term biomonitoring, especially for occupational exposures. However, a spot urine specimen is particularly unreliable because it is subject to large biological variations that necessitate a creatinine excretion correction. Urine-Pb originates from plasma-Pb that is filtered at the glomerular level; thus, according to some authors (Tsaih et al. 1999), urine-Pb levels that are adjusted for glomerular filtration rate may serve as a proxy for plasma-Pb. Hirata et al. (1995) found a better correlation between the concentration of plasma-Pb and urine-Pb than between BPb and urine-Pb for lead workers with low levels of Pb exposure. Manton et al. (2000), using high-precision Pb isotope ratio measurements, found the concentration of urine-Pb to be about 10% of that in whole blood; however, the correlations were not particularly robust. In contrast, correlations with isotopic ratios were excellent. According to Tsaih et al. (1999), cortical bone contributes a mean of 0.43 μg Pb per day excreted in urine, whereas trabecular bone contributes as much as 1.6 μg Pb per day. Cavalleri et al. (1983) observed different Pb kinetics between exposed and nonexposed subjects after the administration of CaNa_2_EDTA. In unexposed subjects BPb levels remained stable even after 5 hr of CaNa_2_EDTA administration. However, plasma-Pb levels in the unexposed group decreased by as much as one half, while urine-Pb increased by a factor of 10. In the Pb-exposed group the same amount of chelation therapy resulted in plasma-Pb levels increasing by a factor of 2, while BPb levels decreased by a factor of 2, with a higher urine-Pb excretion. Thus, it seems that in nonexposed subjects a major contribution for urine-Pb is derived from the Pb fraction in soft tissues that is in equilibrium with that in plasma compartment. We could speculate that the larger the amount of erythrocyte-bound Pb, the weaker the binding forces, and that a significant fraction of Pb is released from red blood cell membranes into plasma and is then filtered by the kidney. Because the amount of Pb excreted is very high, the kidneys are unable to remove it rapidly from the blood stream; this may account for the temporal elevation of plasma-Pb levels.

The availability of reliable urine quality-control materials and reference materials certified for Pb content and participation in external quality assessment schemes for urine-Pb are important factors in assuring the accuracy of analytical results. However, the tendency for urate salts to precipitate out of urine during transit and storage can be a complicating factor in the analysis. Moreover, because only a few studies have examined associations between urine-Pb and other biomarkers, the use of urine-Pb measurements is essentially limited to long-term occupational monitoring programs, monitoring patients during chelation-therapy, and, until very recently, to clinical evaluation of potential candidates for chelation therapy.

Measurement of fecal-Pb content over several days is one possible approach to estimating the overall magnitude of childhood Pb intake. According to Gwiazda et al. (2005), fecal-Pb content should give an integrated measure of Pb exposure/intake from all sources, dietary and environmental, inside and outside the home (by isotopic composition). However, a limitation to the use of fecal-Pb is that the collection of complete fecal samples over multiple days may not be feasible. As stated by Gwiazda et al. (2005), fecal-Pb reflects unabsorbed, ingested Pb plus Pb that is eliminated via endogenous fecal (biliary) routes; interindividual variations in these physiologic processes may show up as variation that is wrongly attributable to environmental Pb exposure.

## Nail Lead

Like hair, nails have many superficial advantages as a biomarker for Pb exposure, especially because specimen collection is noninvasive and simple and because nail specimens are very stable after collection, not requiring special storage conditions. Nail-Pb is considered to reflect long-term exposure because this compartment remains isolated from other metabolic activities in the body (Takagi et al. 1988). Because toenails are less affected by exogenous environmental contamination than fingernails, they have been preferred for Pb exposure studies. Toenails have a slower growth rate than fingernails (up to 50% slower, especially in winter) and thus may provide a longer integration of Pb exposure.

Lead concentration in nails depends on the subject’s age (Nowak and Chmielnicka 2000), but it seems not to depend on the subject’s sex (Rodushkin and Axelsson 2000).

Gulson (1996a) reported high variability in Pb levels measured in the same fingernails and toenails of various subjects, even after rigorous washing procedures; such lack of reproducibility suggests that nail specimens offer only limited scope in assessing exposure to Pb.

## Bone Lead

Because bone accounts for > 94% of the adult body burden of Pb (70% in children) (O’Flaherty 1995), many researchers accept that a cumulative measure of Pb dose may be the most important determinant of some forms of toxicity (cumulative measure means an exposure that is integrated over many years, rather than based on a single BPb measurement) (Landrigan and Todd 1994; Hu et al. 1998). In support of this hypothesis, recent studies have shown that bone-Pb but not BPb is significantly related to declines in hematocrit and hemoglobin among moderately Pb-exposed construction workers and to decreased birth weight and increased odds of clinically relevant hypertension (Gonzalez-Cossio et al. 1997; Hu et al. 1996). According to Hu et al. (1998), other adverse health outcomes likely to be associated with bone-Pb levels include impairment of cognitive performance and growth in children and kidney failure, gout, elevated blood pressure, reproductive toxicity, and adverse cardiovascular events in adults.

As pointed by Hu et al. (1998), two major paradigms relate to skeletal Pb: bone-Pb as an indicator of cumulative Pb exposure (bone-Pb as a repository), and bone-Pb as a source of body burden that can mobilized into the circulation (bone-Pb as a source). Hernandez-Avila et al. (1998) reported a strong association between bone-Pb levels and serum-Pb levels of adults exposed to Pb. That study indicated the potential role of the skeleton as an important source of endogenous, labile Pb that may not be adequately discerned through measurement of BPb levels. The same authors argued that skeletal sources of Pb accumulated from past exposures should be considered along with current sources when exposure pathways are being evaluated. In an attempt to characterize the source of Pb exposure, Gulson et al. (1995) measured the ^206^Pb/^204^Pb isotopic ratios in immigrant Australian subjects, Australian-born subjects, and environmental samples. The immigrant population exhibited Pb isotopic ratios from 17.7 to 18.5, distinct from the ratio in Australian-born subjects (~ 17.0). This difference allowed a distinction to be drawn between current exposure acquired from Australian sources and older bone-stored Pb that was not acquired from Australian sources.

Differing bone types have differing bone-Pb mobilization characteristics. For example, the tibia principally consists of cortical bone, whereas the patella is largely trabecular bone. Pb in trabecular bone is more biologically active than Pb in cortical bone, and trabecular bone has a shorter turnover time. The endogenous contribution of Pb from bone stores is an important health consideration. The O’Flaherty kinetic model can be used to indicate the quantity of Pb delivered from bone as a function of bone turnover and Pb exchange (O’Flaherty 1995). A recent revision of this model (Fleming et al. 1999) suggests that a smeltery worker with a tibia Pb concentration of 100 μg/g can expect a continuous endogenous contribution to BPb of 16 μg/dL. A pregnant woman with a tibia Pb concentration of 50 μg/g can end up with a contribution of 8 μg/dL BPb; this figure does not consider the increased rate of bone turnover associated with pregnancy. Individuals not exposed to Pb in the workplace typically display tibia Pb levels up to about 20 μg/g (Roy et al. 1997).

Over the last decade bone-Pb measurements based on noninvasive *in vivo* X-ray fluorescence (XRF) methods have become increasingly accepted. The technique uses fluorescing photons to remove an inner-shell electron from a Pb atom, leaving it in an excited state. The result is emission of X-ray photons that are characteristic of Pb. Measurements are performed by using one of four kinds of XRF: two involve fluorescence of the K-shell electrons of Pb (K-XRF), and the other two involve fluorescence of the L-shell electrons (L-XRF) (Todd et al. 2002a). Several groups, mainly in North America, have reported the development of *in vivo* measurement systems; the majority have adopted the K-XRF approach based on excitation with a ^109^Cd isotope and backscatter geometry because of its advantages: it provides a robust measurement with a better detection limit and a lower effective (radiation) dose (as compared to L-XRF) (Todd and Chettle 1994). The radiation dose is not a limiting factor in using this technique with humans, as demonstrated by Todd and Chettle (1994).

Calibration is usually performed with Pb-doped plaster-of-Paris phantoms (Todd et al. 2002a). Method accuracy has been evaluated through comparison of XRF data from cadaver specimens with electrothermal atomic absorption spectrophotometry data (Todd et al. 2002b). However, XRF sensitivity and precision for Pb still constitute an analytical challenge. In addition to sample-to-sample reproducibility, XRF can also display a certain amount of imprecision associated with each calculated bone-Pb value (Ambrose et al. 2000). This uncertainty, estimated using a goodness-of-fit statistic from the curve fitting of the background, ranged from 3 to 30 μg/g Pb; clearly, this represents a problem for measurements of low-level Pb namely, young children and nonexposed populations. Another problem inherent to the XRF technique is photon scattering due to overlying tissue or subject movement during the measurement period (Ambrose et al. 2000). Normalization of the Pb signal to the calcium backscatter signal appears to solve this problem. Precision depends on the amount of tissue overlying the bone: the greater the thickness of tissue, the poorer the precision. Todd and Chettle (1994), comparing the K-shell with L-shell precisions with 3 and 6 mm of overlying soft tissue, reported that K-XRF precision worsens by only 5%, whereas L-XRF precision worsens by 49% for greater thickness. The precision of the L-XRF method is much more severely affected by the strong attenuation of the Pb L-shell X rays.

Todd et al. (2001) reported contiguous inhomogeneities in the distribution of Pb toward the proximal and distal ends of the tibia bones. They speculated that the region of lower Pb concentration has lower blood flow in the Haversian canals and, consequently, less Pb available for uptake into bone matrix during bone remodeling (Todd et al. 2001). Trabecular bone has a larger surface area and a greater volume of blood delivered per unit of time compared to cortical bone. In addition there are more active osteons per gram in trabecular bone to accomplish resorption and deposition. Hernandez-Avila et al. (1998) reported that, in individuals with no history to occupational Pb exposure, bone-Pb (in particular trabecular Pb) exerts an additional independent influence on plasma-Pb after control for BPb.

Thus, an appropriate selection of the precise bone type to be analyzed for Pb content must be made before commencing. Moreover, further research on the relationship between various bone-Pb subcompartments and other Pb measures is warranted.

## Tooth Lead

Like bone, teeth accumulate Pb over the long term. However, there is some evidence that teeth are superior to bone as an indicator of cumulative Pb exposure because the losses from teeth are much slower (Maneakrichten et al. 1991). Moreover, deciduous teeth are relatively easy to collect and analyze; exfoliation generally occurs after the age of 6 years. Teeth are also very stable for preservation purposes.

Chronic Pb exposure from mouthing activity in early childhood may be camouflaged by “dilution” effects during periods of rapid skeletal growth in young children and adolescents and may not be detected by a single BPb measurement. However, most published data on tooth-Pb have been based on whole tooth analysis, with no attempt to distinguish among tooth types (different teeth are formed at different ages) or to differentiate the Pb concentration in enamel from that in dentin (enamel contains much more Pb, by mass, than does dentin). The influence of age and/or sex have also not been considered (Brown et al. 2002). Furthermore, use of deciduous teeth is only possible for children over 6 years in age.

Recently, Gomes et al. (2004) proposed a solution to the limitations mentioned above by using an *in vivo* enamel biopsy of children. In this approach superficial minerals are leached from teeth and Pb is determined by electrothermal atomic absorption spectrophotometry. One important drawback to this approach is that, because an accumulation gradient for Pb has not yet been established for enamel, only biopsies of a given depth can be compared to one another. Another issue related to tooth-Pb measurements is whether Pb that accumulates in the first few micrometers of the enamel surface was incorporated posteruptively (e.g., from the mouth, saliva, food) rather than during the period when the tooth was mineralized inside the bone.

An interesting and potentially valuable aspect of tooth-Pb measurements is their capacity to elucidate the history of Pb exposure. Teeth are composed of several distinct tissues formed over a period of several years, and different parts of the tooth can bind Pb at different stages of the individual’s life. Therefore, a section of tooth can yield historical information on the individual’s exposure to Pb. For example, the enamel of all primary teeth, and parts of the enamel from some permanent teeth, are formed *in utero* and thus may provide information on prenatal exposure to Pb. This information could be valuable in improving our understanding of dose–effect relationships for embryonic anomalies, particularly neurotoxic dysfunction. The dentine of the primary teeth provides evidence of exposure during the early childhood years, when hand-to-mouth activity is usually an important contributor to Pb body burden (Gulson 1996b). However, enamel Pb levels may be useful for indirectly estimating the Pb composition of the mother’s bone (Gulson 1996b).

More recently there has been some interest in using laser ablation ICP-MS to examine Pb distribution in tooth profiles. This approach offers spatially resolved measurements of trace element distribution that can be compared to a temporal axis via reference to the neonatal line, enabling researchers to use not only the Pb concentration of the entire tooth but also the specific amount of Pb in each tooth layer, namely, a time line of Pb exposure. Nevertheless, some serious challenges remain before this technique can be fully exploited (Uryu et al. 2003).

## Conclusions

Thus far an impressive body of data has been established based on the use of alternative biomarkers for monitoring exposure to Pb. However, it is still unclear to what extent such data are superior to the information obtained from BPb measurements. Clearly, many of the limitations identified in the foregoing sections must be resolved before alternative biomarkers can be accepted as superior indicators of Pb exposure. At this time BPb measurements are still the most reliable indicator of recent Pb exposure, although serial BPb measurements may offer a better assessment of temporal fluctuations in Pb absorption. If reliable and reproducible plasma-Pb measurements can be obtained, these may offer better correlation with toxic effects. However, we do not yet know what a single plasma-Pb value means, in terms of health effects; in the absence of a normal reference range, the clinical utility for individual assessment is problematic. Further research on this issue is needed, especially for children and adults with low to moderate Pb exposure. Further efforts are also warranted in the further development and continued use of well-established analytical protocols, as well as in the estimation of random and systematic errors. Efforts are needed to create regional reference ranges of nonexposed populations for each biomarker, to acquire data related to long-term and short-term exposures, and to evaluate the influence of nutritional status and ethnicity (genetic polymorphisms).

A critical question that might be asked with respect to an individual’s bone-Pb measurement is what does it mean in terms of health risk or, perhaps, clinical management? To answer this question, we may need to distinguish between bone-Pb measurements in children and pregnant women, namely, those with high bone turnover rate compared to (nonpregnant) adults. In children, bone-Pb may have little effect on BPb levels, but it may help us to estimate the extent to which BPb derives from endogenous sources and the possible contribution to the labile plasma-Pb pool.
